# Comparison of anterior or posterior approach in surgical treatment of thoracic and lumbar tuberculosis: a retrospective case–control study

**DOI:** 10.1186/s12893-022-01611-1

**Published:** 2022-05-10

**Authors:** Jincheng Qiu, Yan Peng, Xianjian Qiu, Wenjie Gao, Tongzhou Liang, Yuanxin Zhu, Taiqiu Chen, Wenjun Hu, Bo Gao, Zhihuai Deng, Anjing Liang, Dongsheng Huang

**Affiliations:** 1 Department of Orthopedics, Sun Yat-Sen Memorial Hospital of Sun Yat-Sen University, Guangzhou, Guangdong China; 2 Musculoskeletal Research Laboratory, Department of Orthopaedics and Traumatology, Faculty of Medicine, The Chinese University of Hong Kong, Hong Kong SAR, China

**Keywords:** Thoracic and lumbar tuberculosis, Anterior, Posterior, Surgical approach

## Abstract

**Background:**

With the widespread use of the posterior surgery, more and more surgeons chose posterior surgery to treat thoracic and lumbar tuberculosis. But others still believed that the anterior surgery is more conducive to eradicating the lesions, and easier to place larger bone pieces for bone graft fusion. We compared the clinical and radiological outcomes of anterior and posterior surgical approaches and presented our views.

**Methods:**

This study included 52 thoracic and lumbar tuberculosis patients at Sun Yat-sen Memorial Hospital from January 2010 to June 2018. All cases underwent radical debridement, nerve decompression, intervertebral bone graft fusion and internal fixation. Cases were divided into anterior group (24 cases) and posterior group (28 cases). Statistical analysis was used to compare the clinical effectiveness, radiological outcomes, complications and other related information.

**Results:**

Patients in the anterior group and the posterior group were followed up for an average of 27.4 and 22.3 months, respectively. There were no statistically significant differences between groups in the preoperative, postoperative and last follow-up VAS score, ASIA grade and Cobb angle of local kyphosis. Moreover, there were no statistically significant differences in the improvement of neurological function, loss of kyphotic correction, total incidence of complications, operative time, intraoperative blood loss and hospital stay between the two groups (*P* > 0.05). But there was greater correction of kyphosis, earlier bone fusion, lower incidence of poor wound healing, less interference with the normal spine and less internal fixation consumables and medical cost in the anterior group (*P* < 0.05).

**Conclusions:**

Both anterior and posterior approaches are feasible for thoracic and lumbar tuberculosis. While for thoracic and lumbar tuberculosis patients with a single lesion limited in the anterior and middle columns of the spine without severe kyphosis, the anterior approach surgery may have greater advantages in kyphosis correction, bone fusion, wound healing, protection of the normal spine, and medical consumables and cost.

## Introduction

According to the Global Tuberculosis Report published by the World Health Organization on 14 October 2021 [1]: in 2020, an estimated 9.9 million people had tuberculosis, and about 1.5 million deaths were attributed to this disease including 214,000 people with HIV.

Bone and joint tuberculosis are the most common types of extrapulmonary tuberculosis, and more than 50% of these are spinal tuberculosis, accounting for about 1–3% of all tuberculosis and more than 40% of all spinal infections [2–4]. Spinal tuberculosis is usually caused by mycobacterium tuberculosis invading the dense blood vessels in the cancellous bone of the vertebral body through blood spread. The lesions usually first occur in the lower front of the vertebral body, and then spread to the center of the vertebral body or the intervertebral disc, causing the destruction of vertebrae and intervertebral disc, forming cold abscess, leading to spinal instability and kyphosis, and spinal cord or caudal equina compression, resulting in neurological dysfunction or even paralysis [2]. About 90% of spinal tuberculosis occurs in the thoracic and lumbar spine, while the cervical and lumbosacral are less affected [5, 6]. Standardized anti-tuberculosis chemotherapy is the most important method for the treatment of tuberculosis. However, when combined with huge abscess, spinal instability, kyphosis, or nerve compression, anti-tuberculosis chemotherapy alone is difficult to achieve satisfied therapeutic results. Therefore, a combination of anti-tuberculosis chemotherapy and surgical treatment with strict indications is generally adopted at present [7].

In recent years, with the widespread use of pedicle screw internal fixation and the development of posterior surgery, more and more surgeons chose posterior surgery to treat thoracic and lumbar tuberculosis [8, 9]. It is believed that the pedicle internal fixation system used in posterior surgery has significant advantages in the recovery of spine stability, the correction of kyphosis, and the maintenance of orthopedic effects [10, 11]. However, some scholars believed that the bone destruction and abscesses of thoracic and lumbar tuberculosis are mostly located in the anterior column or paravertebral column of the spine. Anterior surgery can expose the lesions under direct vision, which is conducive to eradicating the lesions. Furthermore, expose through anterior approach can reduce muscle dissection and avoid damage to the normal posterior column structure. Finally, it is easier to place larger bone pieces for reconstructing through anterior approach [5]. However, the optimal approach for thoracic and lumbar tuberculosis has not been determined. This study aimed to compare the outcomes of the anterior and posterior approach surgery of thoracic and lumbar tuberculosis and to provide reference data for future clinical procedures.

## Materials and methods

### Study design

This is a retrospective case control study. Patients with thoracic and lumbar tuberculosis who underwent surgery at Sun Yat-sen Memorial Hospital of Sun Yat-sen University between January 2010 to June 2018 were included in the present study and were divided into anterior group and posterior group according to the different surgical approaches. Outcomes of patients in both groups were followed up. Each patient signed a consent form for data collection. And this study was approved by the Ethnic Committee of Sun Yat-sen Memorial Hospital.

### Subject selection

The inclusion criteria were as follows: (1) Patients who have been diagnosed with thoracic or lumbar tuberculosis by medical history, clinical manifestations, laboratory examinations, imaging examinations, lesion tissue smears and tuberculosis bacteria culture or postoperative lesion tissue pathological examinations; (2) Patients with single lesion of thoracic or lumbar tuberculosis, and underwent simple anterior or posterior surgery; (3) The patients were completed in our hospital from the first visit to the last follow-up, and the follow-up time was more than 1 year or at least until the bone fusion occurred.

The exclusion criteria were as follows: (1) Patients whose tuberculosis lesions only invaded the cervical or sacral spine or with multiple spinal tuberculosis lesions; (2) Patients who underwent anterior and posterior combined approach surgery; (3) Patients who suffered from other diseases of the thoracic and lumbar spine that affect the clinical efficacy, such as tumors, purulent infections, ankylosing spondylitis, disc herniation, spondylolisthesis, spinal stenosis, etc.; (4) Patients with considerable loss of follow-up data, or follow-up time less than 1 year and without bone fusion.

### Grouping

According to the above inclusion and exclusion criteria, a total of 52 patients with thoracic or lumbar tuberculosis were included in the study, including 29 males and 23 females, with an average age of (42.2 ± 18.0) years old. According to different surgical approaches, they were divided into the anterior group and the posterior group. Among them, 24 patients in the anterior group (12 males and 12 females), aged 41.1 ± 19.3 years, were followed up for an average of 27.2 (13.1–35.3) months; 28 patients in the posterior group (17 males, 11 females), aged 43.2 ± 17.1 years old, were followed up for an average of 22.3 (16.2–38.0) months. The lesions of all 52 patients were located between T4 and L5/S1 intervertebral discs. We assessed the extent of lesion severity based on the number of vertebral bodies and intervertebral discs involved, and the lesions in most cases involved one intervertebral disc and two adjacent vertebral bodies. There were no significant differences in gender, age, follow-up time, lesion location and lesion severity between the two groups (Table 1).

**Table 1:** Preoperative Patient Characteristics

	Anterior (n = 24)	Posterior (n = 28)	Total (n = 52)	*P*
Gender				
Male	12	17	29	0.577
Female	12	11	23	
Age (years)	41.1 ± 19.3	43.2 ± 17.1	42.2 ± 18.0	0.685
Follow-up period (months)	27.2 (13.1–35.3)	22.3 (16.2–38.0)	23.2 (15.5–36.6)	0.964
Lesion location^a^				
T4-T10/11	9	13	22	0.752
T11-L2	11	10	21	
Below L2	8	9	17	
Lesion severity				
Involved vertebral body (n)	2.0 (1.3–2.8)	2.0 (2.0–2.0)	2.0 (2.0–2.0)	0.588
Involved intervertebral disc (n)	1.0 (1.0–1.8)	1.0 (1.0–1.0)	1.0 (1.0–1.0)	0.086

^a^Some lesions were involved in two lesion locations simultaneously

### Study methods

#### Preoperative preparation

All patients without indications of emergency surgery were routinely treated with preoperative anti-tuberculosis chemotherapy for 2–3 weeks, as well as the day of operation. The anti-tuberculosis drugs include isoniazid, rifampicin, pyrazinamide, ethambutol, and streptomycin. We used a four-drug combination anti-tuberculosis regimen including isoniazid and rifampicin (except for drug allergies or intolerances).

#### Surgical procedure

*Anterior approach surgery* The patient was placed in lateral decubitus position after general anesthesia. Thoracoscopy, lateral thoracotomy, retroperitoneal or thoracoabdominal combined approach was used to expose the lesion. Complete debridement and sufficient decompression in front of the spinal cord were performed. The sclerotic bone or suspected necrotic bone around the lesion should be scraped and trimmed to prepare the bone graft bed. Rib or iliac bone of appropriate length was taken as bone graft. Titanium mesh was used or not depended on the degree of bone defect. Anterior single rod or double rods screw internal fixation was performed to restore spinal stability. When the bone destruction of the affected vertebrae was less than 50%, screws could be inserted; otherwise, the screws should be inserted in the adjacent vertebrae of the affected vertebrae. Once the titanium mesh was placed, the ends of the titanium mesh were required to contact with the normal bony endplate. The incision was rinsed with a large amount of normal saline, hydrogen peroxide and iodophor solution of type three in turn. Then 1.5 g of streptomycin or isoniazid powder was sprinkled into the lesions. A closed thoracic drainage tube or common drainage tube was placed, and the incision was closed.

*Posterior approach surgery* After general anesthesia, the patient was placed in a prone position and the lesion was located by C-arm machine. A posterior midline incision was made centered on the lesion. The skin, subcutaneous tissue and myofascia were dissected to expose the lamina and facet joints of corresponding segments, and the thoracic lesion was exposed to the transverse process and even part of the ribs. Generally, pedicle screws were inserted in 1–2 vertebral bodies above and below the lesion. When the lesion was large or the stability of the spine was poor, pedicle screws were inserted in 3 vertebral bodies above and below the lesion. Decompression by laminectomy was performed. The dural sac and nerve roots should be fully exposed. The intercostal nerves and vessels involved were ligated if necessary. Complete debridement was performed to remove tissues such as pus, granulation tissue, dead bone, and residual intervertebral disc. The sclerotic bone or suspected necrotic bone around the lesion should be scraped and trimmed to prepare the bone graft bed. The bone used for bone grafting was obtained from spinous process, lamina or articular process by posterior spinal canal decompression. If not enough, rib or iliac bone was selected. Titanium mesh was used or not depended on the degree of bone defect. If the titanium mesh was placed, the ends of the titanium mesh were required to be in contact with the normal bony endplate. The incision was rinsed with a large amount of normal saline, hydrogen peroxide and iodophor solution of type three in turn. Then 1.5 g of streptomycin or isoniazid powder was sprinkled into the lesions. A closed thoracic drainage tube or common drainage tube was placed, and the incision was closed.

#### Postoperative treatment

The drainage tube was removed when the amount of drainage was less than 50 ml/d. The thoracic closed drainage tube was removed about 48 h after the operation. The Standard anti-tuberculosis chemotherapy was continued, including a four-drug combination of isoniazid and rifampicin (except for drug allergies or intolerances). The course of treatment was 12–18 months, of which 3–6 months was intensification. Erythrocyte sedimentation rate (ESR) and liver function were reviewed weekly during hospitalization and monthly after discharge. Patients were followed up every 3 months postoperative, and their X-ray images were reviewed. Computerized tomography (CT) was performed when needed to further confirm the bone fusion status. After bone fusion, follow-up was done 1–2 times per year.

### Statistics analysis

SPSS 22.0 (IBM, Chicago, IL, USA) was used for data analysis. In statistical description, for continuous variables that conformed to a normal distribution, the mean ± standard deviation was used; if they do not conformed to a normal distribution, the median (interquartile range) was used. The Kolmogorov–Smirnov test was used for the normality test, and the Levene test was used for the homogeneity of variance test. This study involved the comparison of two independent samples. For quantitative data, if the samples followed a normal distribution and the variances were uniform, then the analysis of variance was used. If the above conditions are not met, the rank-sum test (Kruskal–Wallis test) was used. For qualitative data, the comparison of the two groups of binary data and the comparison of the two groups of unordered multi-class data used the chi-square test or the exact probability method, while the comparison of the two groups of ordered multi-class data used the rank-sum test (Kruskal–Wallis test). Differences in the bone fusion rate between different surgical approaches were calculated with log-rank test of Kaplan–Meier analysis. A significance level of 0.05 was adopted.

## Results

### Improvement of clinical symptoms

In terms of clinical symptoms, this study focused on patients' pain symptoms and neurological function, so the VAS score and ASIA grade were used to evaluate the improvement of patients' clinical symptoms. Both surgical approaches were able to achieve pain relief, and the VAS score of last follow-ups were significantly lower than those of preoperative (P < 0.001), but there was no statistical difference between the two groups in the VAS scores of preoperative (P = 0.547), postoperative (P = 0.396) and last follow-up (P = 0.638) (Table 2). In terms of neurological function, there were no patients with ASIA grade A or B in all cases included in this study. Anterior group: 2 cases were grade C preoperative, 1 case improved to grade D and 1 case improved to grade E at the last follow-up; 5 cases were grade D preoperative, 1 case maintained grade D and 4 cases improved to grade E at the last follow-up. Posterior group: 10 cases were grade D preoperative, 1 case maintained grade D and 9 cases improved to grade E at the last follow-up (Table 3). There was no statistical significance in the improving of neurological function between the two groups (Table 4).

**Table 2:** VAS score

	Anterior (n = 24)	Posterior (n = 28)	Total (n = 52)	*P*
Preoperative	6.42 ± 1.59	5.96 ± 2.20	6.17 ± 1.94	0.547
Postoperative	1.50 ± 0.59	1.39 ± 0.88	1.44 ± 0.75	0.396
Last follow-up	0.67 ± 0.96	0.64 ± 1.13	0.65 ± 1.05	0.638
*P*	< 0.001^*^	< 0.001^*^	< 0.001^*^	

^*^P < 0.05, preoperative versus last follow-up

**Table 3:** Changes in the ASIA spinal cord injury scale

	Preoperative	Last follow-up		
		C	D	E
Anterior				
C	2	0	1	1
D	5	0	1	4
E	17	0	0	17
Posterior				
C	0	0	0	0
D	10	0	1	9
E	18	0	0	18

**Table 4:** Improvement of neurological function

Improved grade of ASIA^‡^	Anterior (n = 7)	Posterior (n = 10)	*P*
0	1	1	0.691
1	5	9	
2	1	0	

^*^P < 0.05

^‡^Grade 0 means no improvement; Grade 1 means ASIA improved from C to D, or from D to E; Grade 2 means ASIA improved from C to E

### Comparison of radiological outcomes

**Fig. 1:**
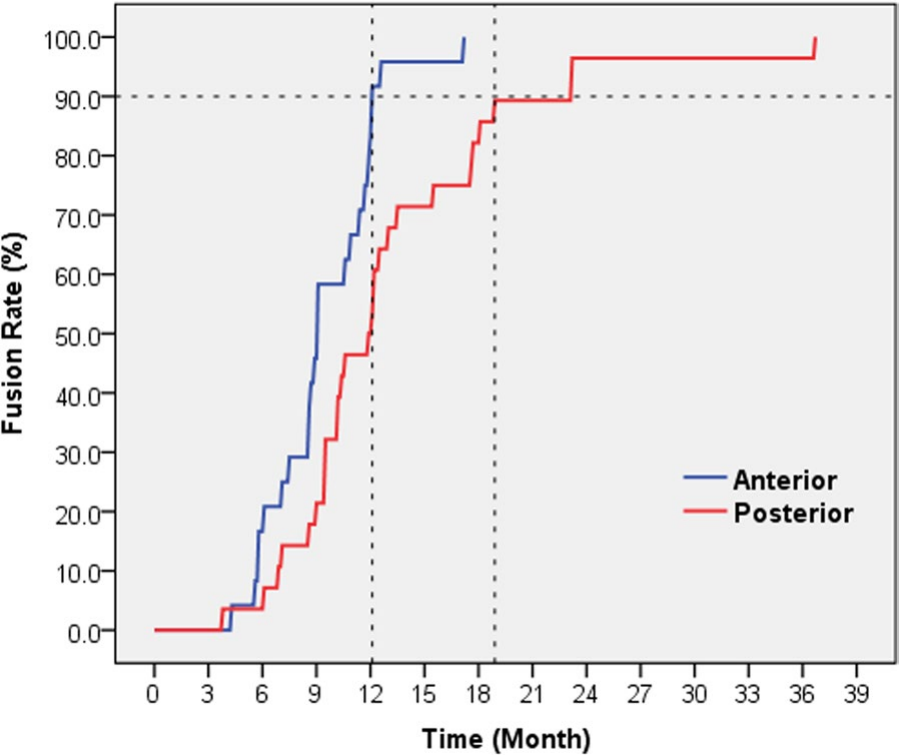
Comparison of bone fusion rate between the two groups. Blue represents the anterior group, and red represents the posterior group. Bone fusion occured earlier in anterior group than posterior group, *P* = 0.011

The majority of cases in this study did not develop severe kyphosis, the local kyphosis Cobb angle of the anterior group: 16.7° preoperative, 5.9° postoperative and 11.2° at the last follow-up; While in the posterior group: 15.1° preoperative, 8.7° postoperative and 12.5° at the last follow-up. There were no statistical differences in the local kyphosis Cobb angle between the two groups preoperative, postoperative and last follow-up. The local kyphosis Cobb angle correction in the anterior group was 10.2°, which was higher than that in the posterior group (6.4°), and the difference was statistically significant (*P* = 0.047). The loss and residual of correction angles were 3.2° and 6.0° in the anterior group, 2.4° and 0.6° in the posterior group, respectively, with no statistical difference between the two groups (Table 5). All patients in this study finally achieved bone fusion, but the time required to reach 90% bone fusion rate was different. It was 12.1 months in the anterior group, significantly earlier than 18.8 months in the posterior group (*P* = 0.011) (Fig. 1).

**Table 5:** Postoperative recovery of local kyphosis Cobb angle

	Cobb Angle^†^ (°)					
Group	Preoperative	Postoperative	Last follow-up	Correction	Loss	Residual
Anterior	16.7 (1.8–33.8)	5.9 (− 2.1–19.1)	11.2 (− 1.9–22.8)	10.2 (3.9–16.5)	3.2 (1.0–5.8)	6.0 (1.7–11.6)
Posterior	15.1 (− 13.2–24.9)	8.7 (− 17.6–19.0)	12.5 (− 17.1–25.1)	6.4 (− 0.3–16.7)	2.4 (0.5–7.9)	0.6 (− 3.3–6.3)
*P*	0.294	0.733	0.733	0.047^*^	0.852	0.070

^*^P < 0.05

^†^ “ + ”means kyphosis, “–”means lordosis

### Complications

**Fig. 2:**
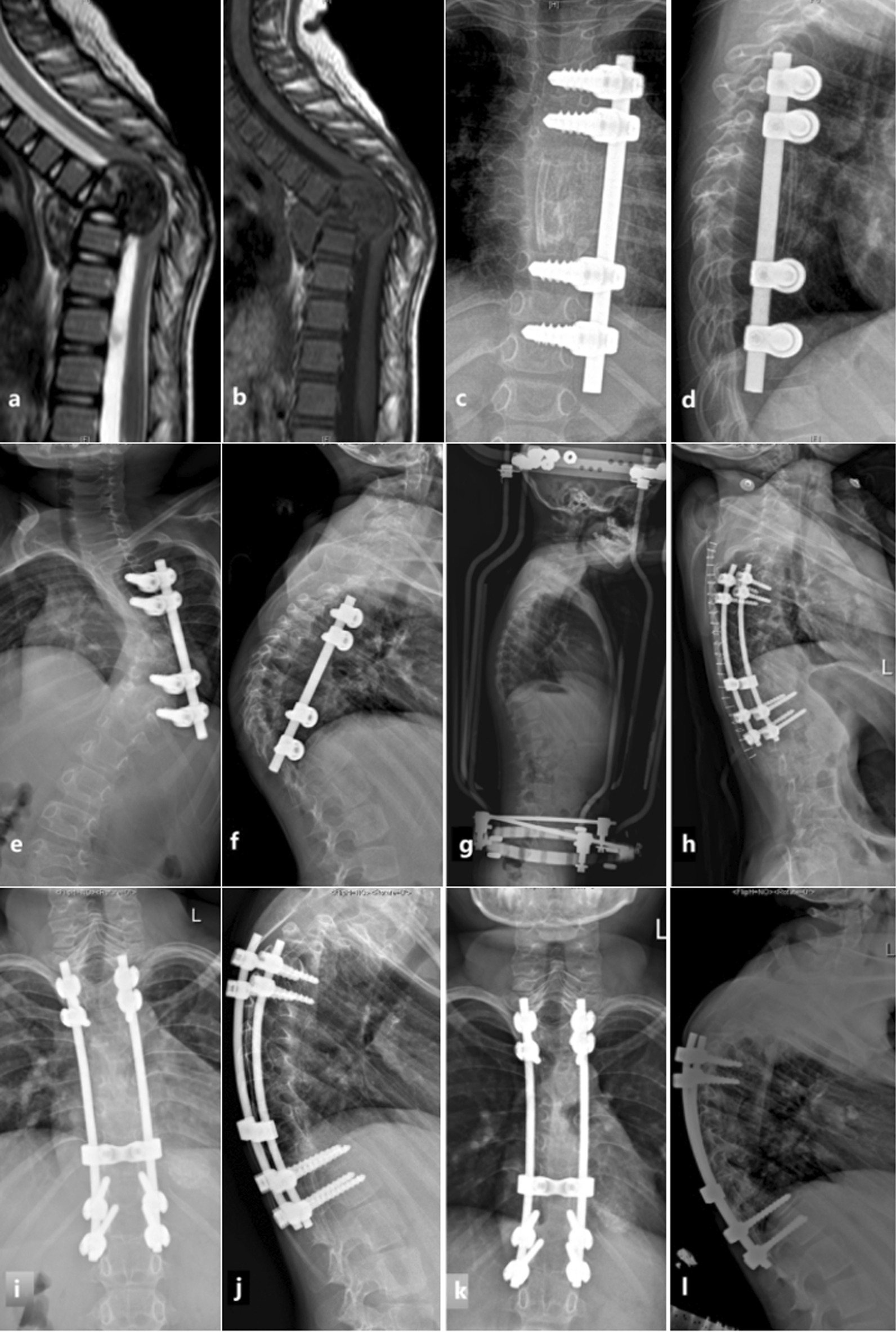
A 4-year-old girl of the anterior group. **a**, **b** Preoperative MR of the patient showed that the T6-8 vertebral body had been severely damaged with massive abscess formation, combined with kyphosis and spinal cord compression; **c**, **d** The patient underwent anterior surgery, where an entire rib was implanted into the lesion to restore vertebral height and kyphosis was significantly corrected; **e**, **f** Three years postoperatively, there was internal fixation displacement, scoliosis, and kyphosis (The anti-tuberculosis regimen was isoniazid + rifampicin + streptomycin for 3 months, and isoniazid + rifampicin for 9 months); **g** 2 months after halo traction; **h** After posterior revision surgery; **i**, **j** 26 months after posterior revision surgery, there was a proximal junctional kyphosis, with a T1-12 Cobb Angle of 88.0°; **k**, **l** 43 months after posterior revision surgery, the proximal junctional kyphosis increased, with a T1-12 Cobb Angle of 110.0°

**Fig. 3:**
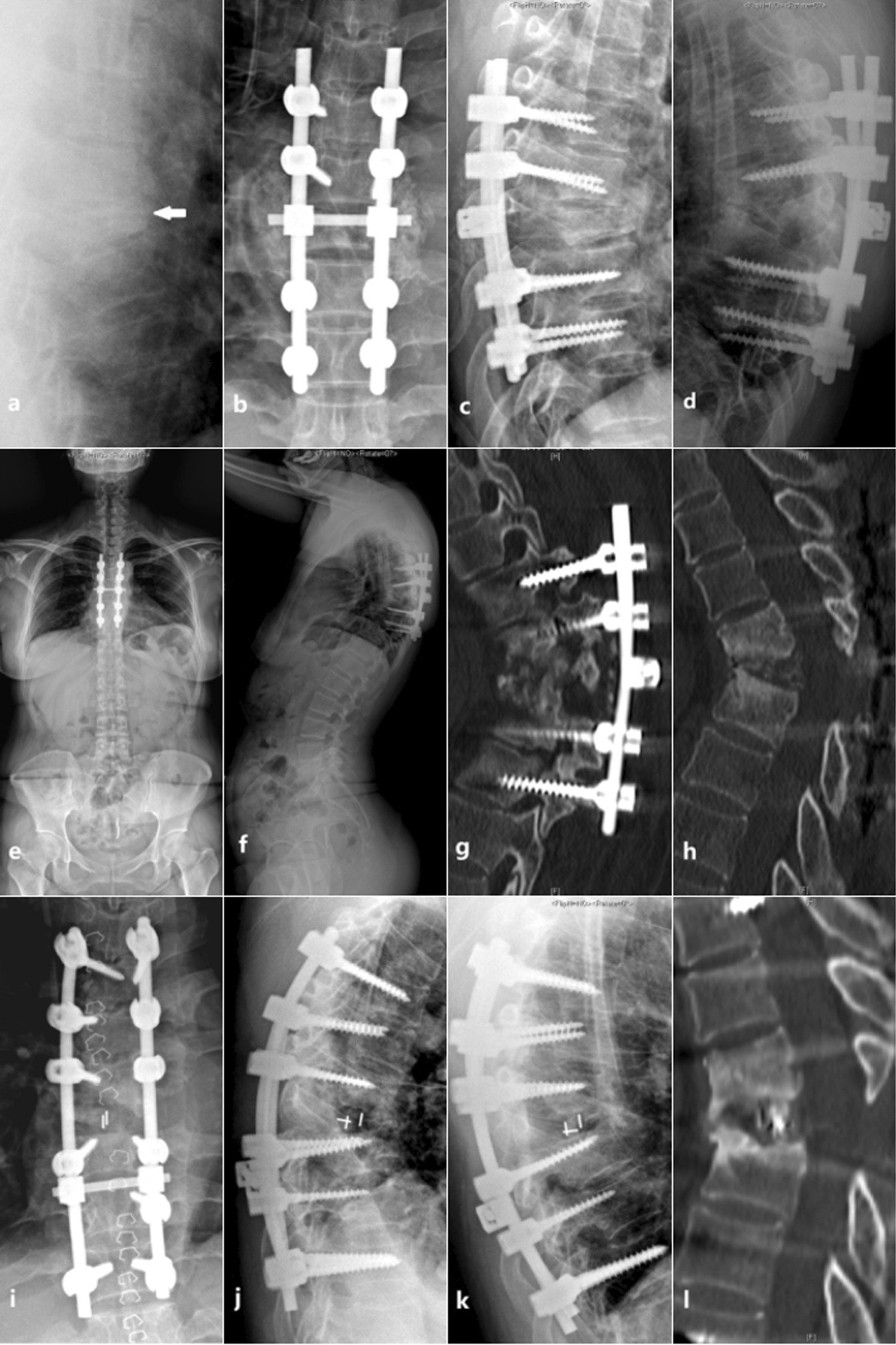
A 36-year-old female patient of the posterior group. **a** Preoperative X-ray of the patient showed that the T7 vertebral body was compressed into a wedge (arrow); **b**, **c** The patient underwent posterior surgery with T5-9 internal fixation; d-h. 29 months after surgery, the patient had a recurrence of tuberculosis with obvious kyphosis, internal fixation release and displacement, destruction and absorption of bone within the lesion, and no bone fusion (The anti-tuberculosis regimen was isoniazid + rifampicin + pyrazinamide + ethambutol for 18 months); **i**, **j** The patient underwent posterior revision surgery, intervertebral cage was implanted, internal fixation was extended by 1 segment to the proximal and distal ends, and the kyphosis was partially corrected (The anti-tuberculosis regimen was changed to isoniazid + rifampicin + pyrazinamide + ethambutol + cycloserine); **k**, **l** 8 months after revision surgery, there were few signs of bone fusion, but a small local kyphosis remained

In this study, there were 6 cases (21.43%) of poor wound healing in the posterior group, while 0 in the anterior group, the difference between the two groups was statistically significant (*P* = 0.025). There were 2 cases (8.33%) with soft tissue abscess or sinus, and 1 case (4.17%) with tuberculosis recurrence in the anterior group. While 5 cases (17.86%) with soft tissue abscess or sinus and 4 cases (14.19%) with tuberculosis recurrence in the posterior group. The posterior group seemed to be associated with higher soft tissue abscess or sinus incidence, and recurrence rates, but none of them reached statistical differences (*P* values were 0.430 and 0.358, respectively). There was 1 case of intercostal neuralgia in the posterior group and 1 case with pain at the bone removal site in the anterior group. There were 3 cases (12.5%) of pneumothorax and 5 cases (20.83%) of pleural effusion occurred in the anterior group, while 2 cases (7.14%) of pleural effusion that occurred in the posterior group. However, there were no significant differences in the incidence of pneumothorax and pleural effusion between the two groups (*P* values were 0.092 and 0.227, respectively), and they were all transient complications in the early postoperative period and were cured after observation or closed thoracic drainage. Internal fixation displacement that occurred in 1 case in each of the two groups (Figs. 2 and 3). There were 2 cases (8.33%) and 1 case (3.57%) of residual kyphosis deformity occurred in the anterior and posterior groups, respectively, but there was no statistical difference (*P* = 0.590). There were 16 cases with complications in total, with a total incidence rate of 30.77%, including 8 cases in the anterior group (33.33%) and 8 cases in the posterior group (28.57%), but there was no statistical difference between the two groups (*P* = 0.769) (Table 6).

**Table 6:** Postoperative complications

Postoperative complications	Anterior (n = 24)	Posterior (n = 28)	Total (n = 52)	*P*
Poor wound healing	0	6(21.43%)	6 (11.54%)	0.025^*^
Soft tissue abscess or sinus	2(8.33%)	5(17.86%)	7 (13.46%)	0.430
Recurrence	1(4.17%)	4(14.29%)	5 (9.62%)	0.358
Intercostal neuralgia	0	1(3.57%)	1 (1.92%)	1.000
Pain at the bone removal site	1(4.17%)	0	1 (1.92%)	0.462
Pneumothorax	3(12.50%)	0	3 (5.77%)	0.092
Pleural effusion	5(20.83%)	2(7.14%)	7 (13.46%)	0.227
Internal fixation displacement	1(4.17%)	1(3.57%)	2 (3.85%)	1.000
Residual kyphosis deformity	2(8.33%)	1(3.57%)	3 (5.77%)	0.590
Cases with complications	8(33.33%)	8(28.57%)	16 (30.77%)	0.769

^*^P < 0.05

### Comparison of surgery and hospitalization related information

The anterior and posterior groups had a mean operative time of 295.8 min and 265.4 min (P = 0.497), a median intraoperative blood loss of 300.0 ml and 325.0 mL (P = 0.732), respectively. The median number of vertebral bodies requiring screw placement in the anterior group was 2, and the number of segments requiring fusion was 3. While in the posterior group, there were 4 vertebral bodies requiring screw placement and 4 segments requiring fusion. The anterior group had less interference with unaffected segments of the spine, and all of these differences were statistically significant (*P* values were < 0.001 and 0.003, respectively) (Table 7). Accordingly, the anterior group required less internal fixation consumables than the posterior group, which was statistically significant. The mean hospital stay was 29.3 days for both the anterior and posterior groups (P = 0.304). The anterior and posterior groups had a median inpatient cost of renminbi (RMB) 72,373 and RMB 91,382 (P = 0.004).

**Table 7:** Surgery and hospitalization related data

	Anterior (n = 24)	Posterior (n = 28)	Total (n = 52)	*P*
Operative time (minutes)	295.8 ± 115.1	265.4 ± 65.4	279.4 ± 92.1	0.497
Intraoperative blood loss (ml)	300.0 (150.0–600.0)	325.0 (140.0–850.0)	300.0 (150.0–675.0)	0.732
Screwed vertebrae (n)	2.0 (2.0–2.0)	4.0 (3.0–4.0)	2.0 (2.0–4.0)	< 0.001^*^
Fusion segment (n)	3.0 (2.0–4.0)	4.0 (3.0–6.0)	4.0 (3.0–4.8)	0.003^*^
Internal fixation consumables				
Screw (n)	4.0 (2.0–4.0)	8.0 (6.0–8.0)	4.0 (4.0–8.0)	< 0.001^*^
Rod (n)	1.5 (1.0–2.0)	2.0 (2.0–2.0)	2.0 (2.0–2.0)	< 0.001^*^
Hospital stay (d)	29.3 ± 7.3	29.3 ± 14.0	29.3 ± 11.3	0.304
Medical cost (RMB)	72,373 (57,709–91,665)	91,382 (75,012–125,089)	86,000 (65,481–106,864)	0.004^*^

^*^P < 0.05

## Discussion

Thoracic and lumbar spine tuberculosis is the most common type of spine tuberculosis, which often leading to paravertebral abscesses, spinal instability, kyphosis and neurological dysfunction [2]. The core of tuberculosis treatment is early, combined, appropriate, regular, and full-course anti-tuberculosis chemotherapy, including spinal tuberculosis [12]. However, simple anti-tuberculosis chemotherapy cannot improve kyphosis and nerve compression secondary to tuberculosis, while timely and appropriate surgical intervention can relieve spinal cord compression, correct kyphosis, restore the stability of the spine and prevent the progression of later deformities. In addition, it may increase the cure rate of tuberculosis and reduce the production of drug-resistant tuberculosis [7]. Anterior and posterior approaches are the most commonly used surgical approaches for treating thoracic and lumbar tuberculosis. In this study, the two approaches of surgery can significantly relieve pain and improve preoperative neurological dysfunction, and there is no significant difference in clinical efficacy between the two approaches, which is consistent with previous research results [5, 13, 14].

Spinal tuberculosis lesions usually invade the anterior and middle columns of the spine, and rarely involve the posterior column. Anterior surgery can directly expose the lesions under direct vision and can remove the lesions more thoroughly. Therefore, it is considered to be the standard surgical procedure for thoracic and lumbar tuberculosis debridement [13]. In addition, anterior surgery is easier for reconstructing with implant larger bone pieces for bone graft fusion [5]. However, some scholars said that anterior surgery might lead to insufficient correction of kyphosis, poor spinal stability, and loss of correction [14]. In contrast, posterior surgery can correct the kyphosis caused by tuberculosis of the thoracic and lumbar spine through many methods. For more minor kyphotic deformities (< 30°), kyphosis correction can be achieved even by compressing and shortening the posterior column alone. For severe kyphosis, various posterior osteotomies can be used to correct the kyphosis. Moreover, the posterior surgery adopts a firm pedicle screw internal fixation system, which can yield a better kyphosis correction and improved stability of the spine [15]. However, in our study, the angle of kyphosis correction in the anterior group was larger than that in the posterior group. Firstly, we endorse the superior ability of posterior approach in kyphosis correction, as it can be achieved with a variety of osteotomies. However, the majority of cases (including the cases in this study) did not develop severe kyphosis and thus did not require osteotomy when posterior surgery was performed. In this situation, correction of kyphosis in posterior surgery is more often achieved by compressing the posterior column of the spine and inserting bone fragments in the anterior and middle columns. In contrast, the anterior approach can expand the intervertebral space and implant a larger bone or titanium mesh to correct the kyphosis by directly restoring the height of the spine.

Many scholars believe that the pedicle is the hardest part of the spine, on the contrary, the vertebral body is dominated by cancellous bone. Therefore, for patients with spinal tuberculosis, the stability of pedicle screw and rod fixation used in posterior surgery is significantly better than that of vertebral body screw and rod fixation used in anterior surgery [10, 16]. We strongly agree with this view, but in our study there were no significant differences in loss and residual of kyphosis correction angle between the two groups. This may be due to the lesions of most patients in this study only involved one intervertebral disc and two adjacent vertebral bodies, which mainly located in the anterior and central columns of the spine. When performing anterior surgery, it did not need to interfere the normal structure of the posterior column. Accompany with the use of anterior internal fixation, it is sufficient to maintain the stability of the spine and the correction angle of the kyphosis. Although it may be less stable than posterior pedicle screw fixation.

The studies by Wang et al. [17] and Zhang et al. [18] showed that the mean bone fusion time and final bone fusion rate of the anterior and posterior approach groups were similar. However, the bone fusion rate is a dynamic index that changes with time, so the mean fusion time and the final bone fusion rate are not adequately to fully reflect the differences in bone fusion condition between the two groups. In this study, we plotted the bone fusion rate-time curve to more intuitively compare the bone fusion condition between the two groups (Fig. 1). Although all patients in the two groups eventually achieved bone fusion in our study, the anterior group was significantly faster. This may due to the larger volume of the bone that implanted in by anterior approach surgery, the better implantation location, and less displacement and bone resorption. In addition, anterior approach surgery removes abscesses more thoroughly and causes less damage to normal soft tissue around the lesion and behind the spine [13, 19]. These factors are all conducive to healing and bone fusion.

Studies have shown that the incidence of postoperative complications in the anterior group is higher than that in the posterior group, especially pneumothorax, pleural effusion and internal fixation-related complications [18, 20]. However, postoperative sinus formation was more frequent in patients who underwent a posterior procedure [21]. In this study, there was no significant difference in the total incidence of complications between the two groups, but we believe that the total incidence of complications sometimes does not reflect the severity of complications between the two groups. In this study, the incidence of pneumothorax and pleural effusion in the anterior approach group was higher than in the posterior approach group. However, these are transient complications after transthoracic approach surgery, and most of them will not prolong the patient’s hospital stay or cause serious consequences, and these complications are easier to deal with. The incidence of poor wound healing, soft tissue abscess or sinus, tuberculosis recurrence in the posterior group was significantly higher than that in the anterior group, and these complications were more serious and more difficult to manage. The higher incidence of such complications in the posterior group may be attribute to the difficulty of posterior surgical debridement and the destruction of the normal structure of the posterior spine. When the lesion is incompletely removed, mycobacterium tuberculosis will drain into the soft tissue behind the spine along with the body position, causing the infection to spread to the posterior spine [22].

Tuberculosis recurrence is an important reason for the failure of internal fixation. In the posterior group of this study, one patient with internal fixation displacement was caused by postoperative tuberculosis recurrence. In the anterior group, there was also 1 case of a 4-year-old girl who suffered from the complication of internal fixation failure. With the growth and development of the spine, the internal fixation shifted and kyphoscoliosis gradually developed. Studies have pointed out that in children with spinal tuberculosis, the kyphosis can continue to progress after surgery until adulthood, especially when simple anterior surgery is performed. If the spine is operated with 360° fusion, the situation may be less severe [23]. Most scholars believe that posterior surgery requires fixation of 2 vertebrae at the proximal and distal ends of the lesion [24, 25], with an average of 5 segments fused [4]. While the anterior group has less interference with the normal spine, it requires less internal fixation equipment and treatment costs. The results of this study were consistent with it. There were no significant differences between the two groups in the operative time, intraoperative blood loss and hospital stay.

However this study also has some limitations, the lesions of most cases only involved one disc and adjacent vertebral bodies without severe kyphosis, so that the superiority of posterior approach surgery did not well demonstrate. It is undeniable that posterior pedicle fixation is better than anterior vertebral fixation, so the braces were needed for some patients with a larger lesion in the anterior group. In addition, although anterior surgery is safe for the spinal cord, the risk of large blood vessel damage is higher than that of posterior surgery [17, 26]. Finally, the anterior approach is relatively unfamiliar for most spine surgeons, thus requires a long learning curve and a higher level of surgical skill. Therefore, the choice of surgical approach for each patient needs to be comprehensively considered, and the appropriate surgical approach should be selected individually.

## Conclusion

Both anterior and posterior surgery can effectively treat thoracic and lumbar tuberculosis. There were no significant differences between the two surgeries in terms of pain relief, nerve function improvement, bone fusion rate, total incidence of complications, operation time, intraoperative blood loss and hospital stay. While for thoracic and lumbar tuberculosis patients with lesions only involve one intervertebral disc and adjacent vertebral bodies without severe kyphosis, the anterior approach may have greater advantages in kyphosis correction, bone fusion speed, wound healing, soft tissue and normal spine protection, medical consumables and cost.

## Data Availability

The datasets generated and analyzed during the current study are not publicly available due hospital property and patient privacy, but are available from the corresponding author on reasonable request.

## Abbreviations

ESR: Erythrocyte sedimentation rate
CT: Computerized tomography
VAS: Visual analogue scale
ASIA: American Spinal Injury Association impairment scale
